# Insecticide resistance management and industry: the origins and evolution of the Insecticide Resistance Action Committee (IRAC) and the mode of action classification scheme

**DOI:** 10.1002/ps.6254

**Published:** 2021-01-28

**Authors:** Thomas C Sparks, Nicholas Storer, Alan Porter, Russell Slater, Ralf Nauen

**Affiliations:** ^1^ Corteva Agriscience Indianapolis IN USA; ^2^ Alan Porter Associates Ltd Troon UK; ^3^ Syngenta Crop Protection Basel Switzerland; ^4^ Bayer AG, Crop Science Division, R&D Monheim am Rhein Germany; ^5^ Agrilucent LLC Greenfield IN USA

**Keywords:** insecticide resistance, resistance management, insecticide mode of action, crop protection, sustainable agriculture

## Abstract

Insecticide resistance is a long‐standing problem affecting the efficacy and utility of crop protection compounds. Insecticide resistance also impacts the ability and willingness of companies around the world to invest in new crop protection compounds and traits. The Insecticide Resistance Action Committee (IRAC) was formed in 1984 to provide a coordinated response by the crop protection industry to the problem of insecticide resistance. Since its inception, participation in IRAC has grown from a few agrochemical companies in Europe and the US to a much larger group of companies with global representation and an active presence (IRAC Country Groups) involving an even wider array of companies in more than 20 countries. The focus of IRAC has also evolved from that of defining and documenting cases of insecticide resistance to a pro‐active role in addressing insecticide resistance management (IRM) providing an array of informational and educational tools (videos, posters, pamphlets) on insect pests, bioassay methods, insecticide mode of action and resistance management, all publicly available through its website (https://irac-online.org/). A key tool developed by IRAC is the Insecticide Mode of Action (MoA) Classification Scheme, which has evolved from a relatively simple acaricide classification started in 1998 to the far broader scheme that now includes biologics as well as insecticides and acaricides. A separate MoA Classification Scheme has also been recently developed for nematicides. The IRAC MoA Classification Scheme coupled with expanding use of MoA labeling on insecticide and acaricide product labels provides a straightforward means to implement IRM. An overview of the history of IRAC along with some of its notable accomplishments and future directions are reviewed. © 2021 The Authors. *Pest Management Science* published by John Wiley & Sons Ltd on behalf of Society of Chemical Industry.

## Graphical abstract

Global IRAC country groups

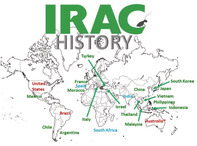

## 1 INTRODUCTION

**Figure 1:**
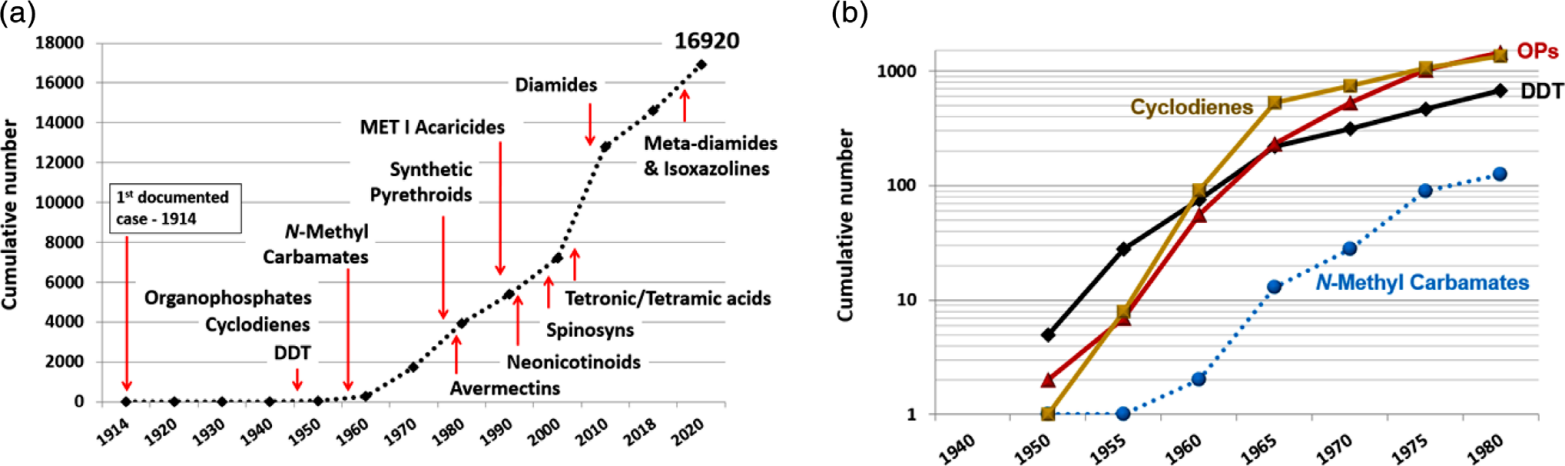
(A) Number of cases of insecticide resistance per decade and approximate dates of introduction for selected major classes of insecticides and acaricides. (B) Cumulative number of cases of resistance to selected insecticides/classes 1940–1980. Resistance cases based on data from the ARPD.^65^ (Mota‐Sanchez and Wise 2020).

The first documented case of insecticide resistance was published more than a 100 years ago.^1^ Insecticide resistance remained an infrequent phenomenon until the advent of synthetic organic insecticides in the 1940s and 1950s (Fig. 1).^2^, ^3^, ^4^, ^5^ With the introduction and consequent expanding use of synthetic organic insecticides such as DDT, the cyclodienes and organophosphates (OPs), there was a rapid, nearly exponential increase in the numbers of cases of insecticide resistance (Fig. 1). The introduction of the *N*‐methyl carbamates in the mid‐1950s (Fig. 1(A)) added a new class of insecticides for which resistance was also observed in subsequent years (Fig. 1(B)). The advent of resistance to the limited number of insecticide classes resulted in control failures for a range of crops, in turn giving rise the concept of integrated control,^6^, ^7^ resistance management,^8^, ^9^, ^10^, ^11^ and the search for new classes of insecticides.

Since the 1950s and 1960s insecticide resistance has come into prominence around the globe as a key factor impacting the use and efficacy of a wide range of existing and new compounds for the control of insect and mite crop pests as well as vectors of human diseases.^2^, ^3^, ^4^, ^5^, ^7^, ^9^, ^12^, ^13^, ^14^, ^15^, ^16^ Insecticide resistance is also an important driver in the search for new insecticides, especially those with new modes of action.^17^, ^18^, ^19^, ^20^, ^21^, ^22^ Within the crop protection industry, insecticide resistance was recognized as a concern as early as the late 1950s to early 1960s.^23^ The early industry response most often involved simply finding and using a different insecticide. Frequently the replacement products were in the same class of chemistry^24^ since there were few distinct classes of insecticides available during this time.^22^, ^25^ However, in some instances recommendations from industry scientists included specific resistance mitigating measures such as moderation of use, alternation (rotation) of insecticides from different classes, and incorporation of biological control measures.^17^, ^23^, ^26^, ^27^

Nearly 40 years ago, increasing pesticide resistance led to the recognition that a coordinated effort among the major crop protection companies was needed to proactively address many of the problems related to resistance, including the overuse of ineffective crop protection compounds, increasing yield loss, increasing costs to growers, and the loss of valuable products due to overuse. One key result was the formation of cross‐industry resistance action committees composed of company technical experts, often advised by independent and university scientists. The Insecticide Resistance Action Committee (IRAC) was formed in 1984, along with its sibling organizations, the Fungicide Resistance Action Committee (FRAC) in 1981, and the Herbicide Resistance Action Committee (HRAC) in 1989. IRAC provides one of the industry's most important and longest running efforts to proactively address insecticide resistance. Herein we provide a short history of IRAC and its evolution over the past 35‐plus years.

## 2 ORIGINS OF IRAC

The major issues with insecticide resistance and the associated large scale control failures, especially in relation to cotton and then available cotton insecticides (e.g. DDT, cyclodienes, OPs, *N*‐methyl‐carbamates) during the late 1960s and early 1970s^7^, ^14^ led to greater adoption of integrated control/integrated pest management (IPM) programs. Based on the hard lessons from the prior two decades, there were proposals for cooperation among crop protection companies for resistance monitoring and the more strategic use of the newly developed synthetic pyrethroid insecticides being commercialized in the late 1970s.^28^, ^29^, ^30^ Multiple crop protection companies had developed or licensed the new synthetic pyrethroid insecticides and were in the process of deploying these new insecticides into the many of the same markets (e.g. cotton). Thus, the concept of a coordinated effort to monitor for resistance came about as a means to minimize overuse and prevent or at least delay the development of resistance.^31^, ^32^ In 1979, the Pyrethroid Efficacy Group (PEG) was established in the US,^31^, ^32^, ^33^ with participation of eight crop protection companies involved in pyrethroid manufacturing.^31^ The principal goals of the PEG were to (i) provide technical advice to researchers, growers and governments on pyrethroid resistance problems and facilitate interactions between these groups, (ii) understand the causes of field failures, and (iii) sponsor research on pyrethroid resistance.^28^, ^29^ To this end, monitoring studies were conducted,^34^ along with research into pyrethroid resistance mechanisms.^29^ Later, PEG became a an IRAC subcommittee (1985).^28^, ^29^

**Figure 2:**
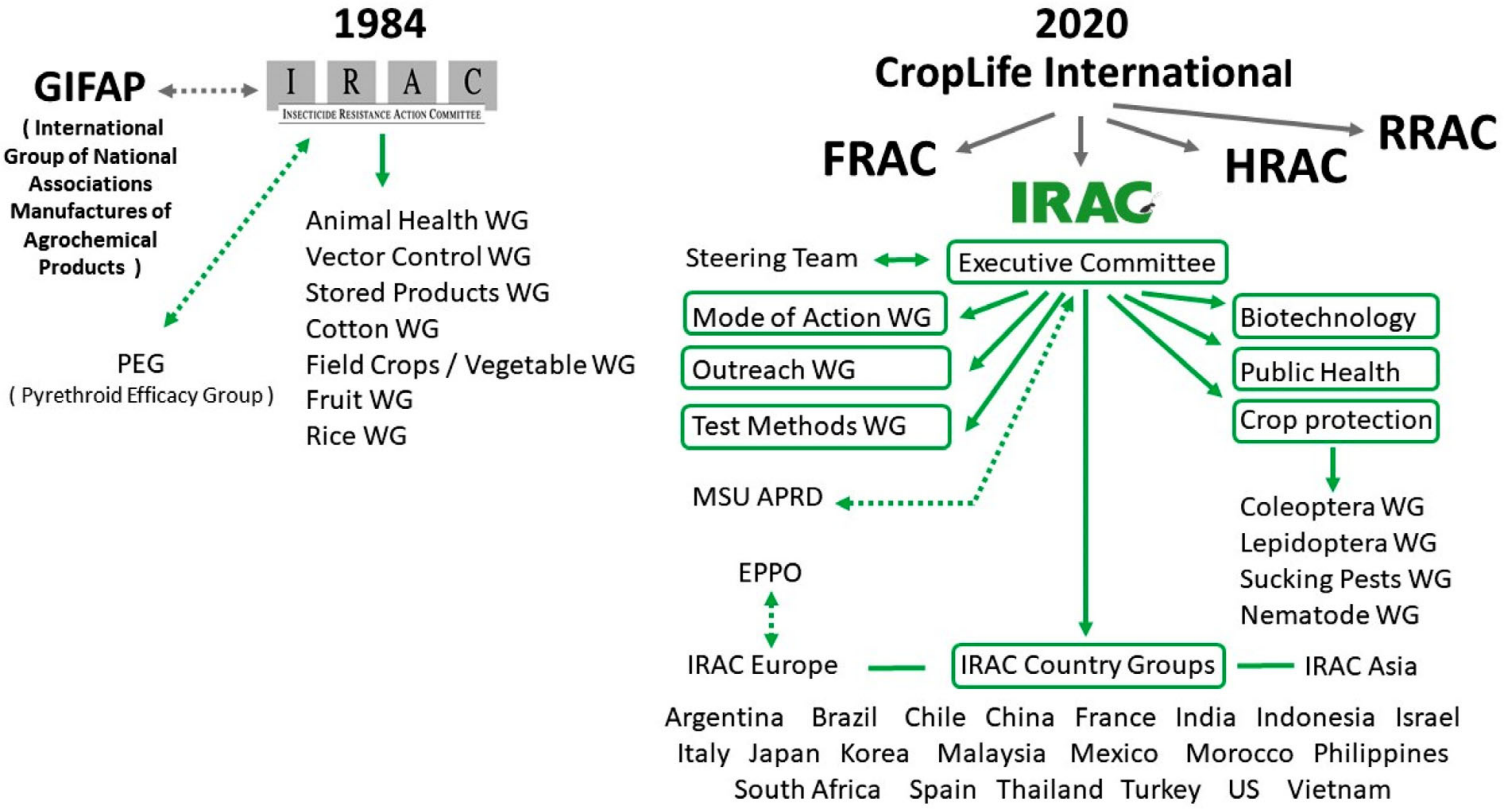
Initial IRAC organization (1984) *versus* current (2020) including working groups (WG). Information from Jackson 1986, IRAC 2020.^31^, ^44^ Dotted lines indicate informal linkages between IRAC and other external organizations. MSU APRD – Michigan State University, Arthropod Pesticide Resistance Database.^65^ EPPO, European and Mediterranean Plant Protection Organization; FRAC, Fungicide Resistance Action Committee (1981); HRAC, Herbicide Resistance Action Committee (1989); RRAC, Rodenticide Resistance Action Committee (1990).

In light of the heightened awareness of resistance issues and as a follow‐up to the PEG, several international technical committees were established by the International Group of National Associations of Manufacturers of Agrochemical Products [Groupement International des Associations Nationales de Fabricants de Produits Agrochimiques] (GIFAP). One of the goals of the GIFAP sponsored technical committees (e.g. IRAC) (Fig. 2) was to provide guidance on technical and scientific matters related to pesticide resistance. The GIFAP (later to become the Global Crop Protection Federation in 1996, and then CropLife International in 2001) was formed in 1967 as the international voice of crop protection associations. GIFAP, and its resistance technical committees, were also advisory bodies to the United Nations organizations; the World Health Organization (WHO) and the Food and Agriculture Organization (FAO).^35^

**Figure 3:**
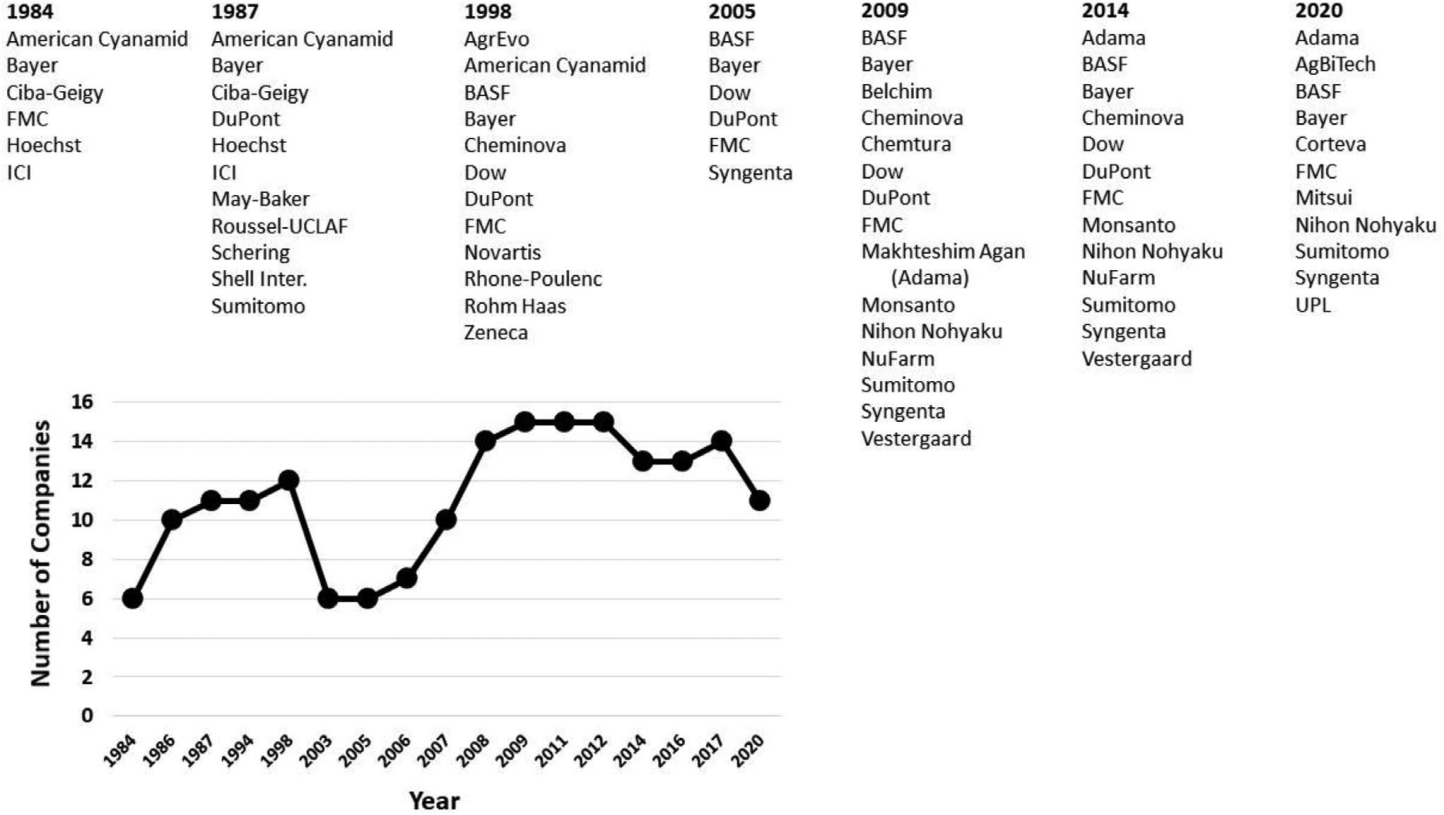
Company numbers and composition of IRAC as a function of time.

**Figure 4:**
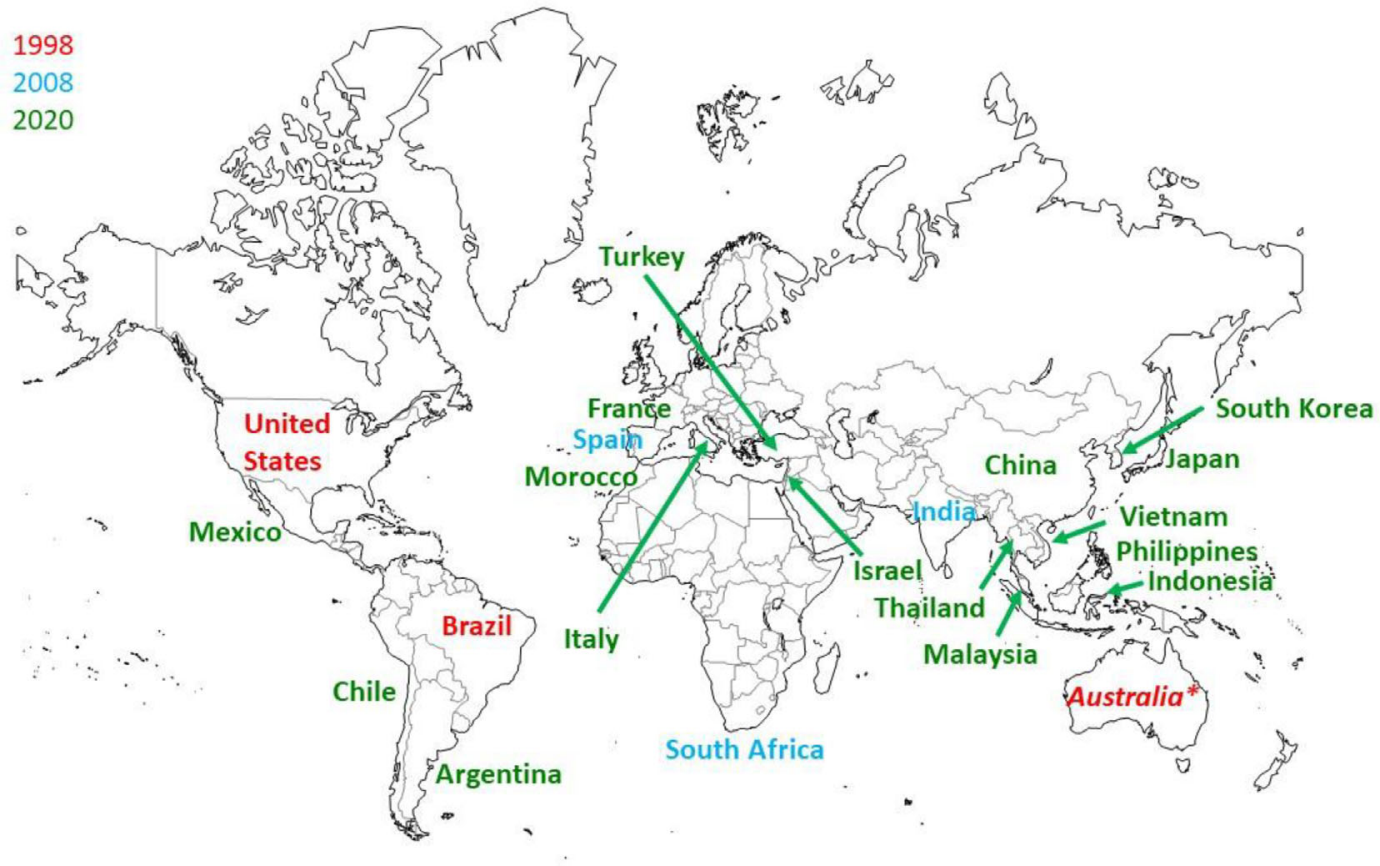
The global expansion of IRAC country groups as a function of time. 1998 (Brazil, US, Australia*), 2008 (addition of India, South Africa, Spain), 2020 ‐ all countries currently listed. Two other IRAC regional groups (not shown) are IRAC Asia and IRAC Europe. *IRAC Australia is linked to CropLife Australia, and not directly to IRAC international ‐ included here for informational purposes.

As initially (1984) formed, IRAC was composed of representatives from six of the leading R&D‐based agrochemical companies at that time (Fig. 3). Over the next 35 years company participation in IRAC has varied (Fig. 3) as companies and the agrochemical industry has gone through mergers and consolidation,^21^, ^22^, ^36^, ^37^, ^38^, ^39^, ^40^ which continues to impact the industry and IRAC (Fig. 3). However, IRAC has always sought to represent the vast majority of the R&D‐based crop protection companies. Since 2009 company membership has expanded to include a broader array of companies from around the globe including all of the major crop protection R&D companies currently in the US and Europe (Germany & Switzerland), and an expanding representation from Japan and the rest of the world (Australia, India, Israel) (Fig. 3). The companies currently participating in IRAC represent approximately 81% of the global market (2018 sales) for crop protection compounds.^40^, ^41^ In addition, to the current 11 member companies of IRAC International, other companies and local experts are members of the IRAC Working Groups and there is an expanding array of Country Teams (Figs 2, 4), further extending the participation in IRAC and its impact. Thus, IRAC is well situated to educate and guide insecticide resistance management programs.

## 3 IRAC OPERATIONS AND FUNCTIONS

### 3.1 Goals and focus

The aim of IRAC when established in 1984 was the identification of resistance and the provision of solutions to resistance problems in the field.^17^, ^31^, ^42^ Additional broad goals included the development of resistance monitoring methods, including those suitable for use in the field, the gathering and sharing of resistance data within the industry, coordination of efforts within industry, and in cooperation with non‐industry scientists in the development of insecticide resistance management (IRM) concepts to prolong the life of insecticides, and the preparation of educational materials regarding insecticides and IRM.^17^, ^24^, ^31^, ^32^, ^43^ The intent behind these goals remain today with IRAC's mission defined as (i) Facilitate communication and education on resistance to insecticides and insect‐resistant traits, and (ii) Promote and facilitate development and implementation of resistance management strategies to maintain efficacy and support sustainable agriculture and improved public health. To achieve these goals, company representation consists of technical experts in applied entomology, insect toxicology, and biochemistry. IRAC operates under the CropLife International anti‐trust guidelines to ensure observance and compliance with all applicable antitrust laws.

### 3.2 IRAC organization and working groups

The leadership and operation of IRAC is shared responsibility among the representative scientists from the member companies. IRAC Chairs are elected from among membership and has rotated among the member companies over the years. The primary activities of IRAC are divided among a number of WGs, with overall oversight by the Executive Committee, which consists of the lead representative from each of the member companies. Since 1999 IRAC has employed a coordinator to oversee some of the day‐to‐day operations and ensure consistency over time as member companies and individual representatives have changed.

Initially the WGs were focused on specific crops (e.g. cotton, rice, fruit, *etc*.) of global interest, as well as stored products, animal health and insect vectors of human diseases (Fig. 2). Also, as initially envisioned, WGs or Task Teams were typically established to bring focus on particular tasks (see example work products below) or areas of interest, and once that task is completed, the Task Team was disbanded. However, in many cases the subject of a particular WG is of long‐term importance and these WGs have *de facto* become permanent. In some cases, the role (and name) of the WG has evolved to encompass broader topics and issues. For example, the Codling moth WG eventually expanded into the larger, more encompassing Lepidoptera WG, that also absorbed the Diamide WG, since the issues and needs required a broader focus. Thus, the crop specific WGs were ultimately replaced by more pest specific (Lepidoptera, Coleoptera, Sucking Pests) WGs (Fig. 2), all tied to the umbrella of Crop Protection (Fig. 2).^44^ The Animal Health and Vector WGs became the broader Public Health WG (Fig. 2). As the structure of IRAC continued to evolve, some topics (e.g. genetically engineered crops with insect protection traits) that were initially addressed by a focal point that then were expanded to a WG as the needs, interest and importance grew. At present the three primary areas of focus for IRM are Plant Biotechnology, Crop Protection, and Public Health (Fig. 2),^44^ all addressed by one or more WGs.

#### 3.2.1 IRAC plant biotechnology team

IRM for insect‐protected genetically modified (GM) crops, and the unique considerations for non‐Bt crop refuges, were originally represented at IRAC via a single focal point, and individual country‐specific industry groups addressed needs on a country‐by‐country basis. For refuge‐based IRM, strong coordination among the crop developers in establishing guidelines and achieving grower implementation is paramount. With the rising importance of GM crops globally, IRAC established the Plant Biotechnology Team (Fig. 2) in 2007 to promote consistency in the application of the scientific considerations and to coordinate communication within the crop developer companies and with external stakeholders. The IRAC Biotech team has played a key role in educating regulators and policy makers on refuge‐based IRM and the importance of pyramided traits (more than one trait in a plant that is active against the same key target pests), while ensuring guidelines and regulations are realistic and practical. Bringing IRM for GM crops into the IRAC community has created new opportunities for IRM programs that integrate the insect‐protected traits with crop protection chemistry to protect durability of both components of the pest management system.^45^

#### 3.2.2 IRAC public health team

From its inception, IRAC had a public health focus in the form of two WGs; the Animal Health WG and the Vectors WG (Fig. 2).^31^ The Vectors WG, also known as the Public Health and Vectors.^17^, ^24^, ^32^ was primarily focused on mosquitoes and providing input to the WHO. The activities of the Animal Health WG ended by 1998, while the Vector WG was active until the early 2000s. The Public Health Team was re‐established in 2006 to continue the work initiated by the previous IRAC Vector Control Group and has the extended remit covering hygiene pests as well as vectors. Major efforts of the team focused on the preparation of educational material such as resistance test method summaries, posters covering IRM in mosquitoes, houseflies, cockroaches and bedbugs, as well as a condensed version of the IRAC MoA classification for mosquito disease vectors. Other efforts have focused on liaison with key groups working in the vector control area like WHO, Bill and Melinda Gates Foundation (BMGF) and the Innovative Vector Control Consortium (IVCC). A key IRAC publication ‘Prevention and Management of Insecticide Resistance in Vectors and Pests of Public Health Importance’ was first published with inputs from these groups in 2007 and an updated edition was published in 2011.^46^ A mini version of this comprehensive booklet concisely covering important public health specific IRM aspects was published in 2011 in two different languages, English and French.

#### 3.2.3 Outreach WG

Another important WG, established in 1999, is the Outreach WG (formerly Communication & Education) (Fig. 2). The growth of IRAC in the 1990s increased the emphasis and activities regarding the development and dissemination of information and educational materials (posters, booklets, videos, *etc*.) related to resistance management. The Outreach WG was formed to oversee these activities in coordination with the other WGs. Further, as a means to provide easy access to information on IRM and the associated activities of IRAC, the Outreach WG established the IRAC website.^47^ The IRAC web‐site (https://irac-online.org/) managed by the IRAC Coordinator and the Outreach WG, now features an array of information on more than 45 different pest insects (Pest pages) across 12 crops, videos on insecticide MoA, resistance and IRM (14 languages) and training modules on insecticide resistance and MoA.

Several years ago, the Outreach WG chartered the development of a mobile phone app for the insecticide MoA classification scheme^4^ that has gone through several iterations and upgrades. All of this information is freely available via the IRAC website. Additionally, since 2004, the Outreach WG has also overseen the publishing of a regular electronic IRAC newsletter (eConnection) to provide external stakeholders with an overview of emerging resistance issues and IRM activities.^48^, ^49^ Interested individuals can sign up to receive the eConnection newsletter through the IRAC website.

#### 3.2.4 IRAC country and regional groups

While resistance management principles are developed at the global level, implementation relies on regional and local action, often driven by needs related to specific crops and pests. As a means to bring greater focus on IRM at the country or regional level, over the years a number of IRAC country groups have been established (Figs 2, 4). Some of these country groups have been short‐lived addressing a particular need at the time, while others have been functioning for many years (e.g. Brazil, South Africa, Spain, US) (Fig. 4). In some cases a particular country group was established, faded, and then re‐established, in others the country group was established by a merger of interests between an external group and an IRAC group, as exemplified by IRAC US established in 1994 by the merger of PEG US and the IRAC Cotton US. Currently, several informal country resistance groups that initially formed to develop local strategies to manage resistance in Lepidoptera to diamides are now becoming formalized as new IRAC Country Groups, resulting in more than 20 IRAC country groups (Figs 2, 4) spanning the globe. In addition, there are also two regional IRAC groups, IRAC Asia and IRAC Europe. IRAC Europe provides an informal linkage to European organizations such as the European and Mediterranean Plant Protection Organization (EPPO).

## 4 EXAMPLES OF IRAC WORK PRODUCTS AND ACCOMPLISHMENTS

IRAC and its associated WGs and task teams are responsible for generating information and publications that facilitate improvements to resistance management practices by companies, extension services, crop consultants, and end‐users. Work products are generally developed by WGs and finalized with approval by the Executive Committee. Some examples of IRAC's work products and the associated impact are highlighted below.

### 4.1 MoA classification, MoA labeling and IRM

One of the fundamental principles of resistance management is avoiding the repeated use of insecticides that have the same mode of action to treat the same pest population. The IRAC MoA classification scheme (Table 1)^4^, ^5^, ^48^, ^49^, ^50^ has become one of the most important initiatives and work products from IRAC, enabling insecticide users to understand which insecticides have the same or different MoAs. The MoA Classification Scheme is the preeminent global insecticide MoA resource, providing a simple, straightforward approach to the selection of insecticides and acaricides for alternation/rotation in IRM protocols.^4^, ^5^, ^49^

**Table 1:** IRAC MoA Classification Scheme 2020 (v9.4) *vs* Original IRAC Acaricide Classification (1988)

2020	2020 (V9.4)			1988
IRAC Grp	IRAC subgroup/exemplifying active	Targeted physiology*	Primary site of action/MoA	IRAC Acaricide Groups
1	1A Carbamates	NM	Acetylcholinesterase Inhibitors	—
	1B Organophosphates (OPs)			—
2	2A Cyclodienes	NM	GABA‐gated chloride channel antagonist	—
	2B Fiproles			—
3	3A Pyrethroids	NM	Voltage‐gated sodium channel modulators	D Pyrethroids
	3B DDT & analogs			—
4	4A Neonicotinoids	NM	nAChR competitive modulators	—
	4B Nicotine			—
	4C Sulfoximines			—
	4D Butenolides			—
	4E Mesoionics			—
5	Spinosyns	NM	nAChR allosteric modulators–Site 1	—
6	Avermectins & milbemycins	NM	Glutamate‐gated chloride channels ‐ allosteric modulators	—
7	7A Juvenoids	GD	Juvenile hormone receptor agonists	—
	7B Fenoxycarb			—
	7C Pyriproxyfen			—
8	8A Alkyl halides	UN/NS	Multi‐site	—
	8B chloropicrin		Multi‐site	—
	8C Fluorides		Multi‐site	—
	8D Borates		Multi‐site	—
	8E Tartar emetic		Multi‐site	—
	8F Methyl isothiocyanate generators		Multi‐site	—
9	9B Pyridine azomethine derv.	NM	Chordotonal organ	—
	9D Pyropropenes			—
10	10A Hexathiazox	GD	Mite growth inhibitors	B Hexathiazox B Clofentezine
	10B Oxazoles	GD		—
11	11A *Bacillus thuringiensis (Bt)*	MG	Midgut membrane	—
	11B *Bacillus sphaericus*			—
12	12A Diafenthiuron	RSP	Inhibitors of ATP synthase	—
	12B Organotin miticides			A Organotins
	12C Propargite			H Propargite
	12D Tetradifon			F Tetradifon
13	Pyrroles, Dinitrophenols, Sulfuramid	RSP	Oxidative phosphorylation ‐ uncouplers	K Dinobuton
14	Nereistoxin analogs	NM	nAChR channel blockers	—
15	Benzoylureas	GD	Chitin synthesis inhibitor	—
16	Buprofezin	GD	Chitin synthesis inhibitor	—
17	Cyromazine	GD	Moulting disruptors, dipteran	—
18	Diacylhydrazines	GD	Ecdysone receptor agonist	—
19	Formamidines	NM	Octopamine receptor agonist	G Amitraz
20	20A Hydramethylnon	RSP	MET III inhibitors	—
	20B Acequinocyl			—
	20C Fluacrypyrim			—
	20D Bifenazate			—
21	21A MET I inhibitors	RSP	MET I inhibitors	—
	21B rotenone			—
22	22A Oxadiazines	NM	Voltage gated sodium channel blocker	—
	22B Semicarbazone			—
23	Tetronic/tetramic acids	GD	Inhibitors of ACCase	—
24	24A Phosphine	RSP	MET IV inhibitor	—
	24B Cyanide			—
25	25A β‐Ketonitrile derivatives	RSP	MET II inhibitors	—
	25B Carboxanilides			—
28	Diamides	NM	Ryanodine receptor	—
29	Flonicamid	NM	Chordotonal org. Mod. Undefined target site	—
30	Meta‐diamides & isoxazolines	NM	GABA‐gated chloride channel allosteric modulators	—
31	Granuloviruses (GVs)/Nucelopoly hedroviruses (NPVs)	MG	Midgut membrane	—
32	GS‐omega/kappa HXTX‐HV1A peptide	NM	nAChR allosteric modulators –Site II	—
UN	Azadirachtin	UN/NS	Unknown	—
	Benzoximate	UN/NS	Unknown	J Benzoximate
	Bromopropylate	UN/NS	Unknown	C Bridged diphenyl cpds
	Chinomethionat	UN/NS	Unknown	I Quinomethionate
	Dicofol	UN/NS	Unknown	—
	Lime sulfur	UN/NS	Unknown	—
	Pyridalyl	UN/NS	Unknown	—
	Sulfur	UN/NS	Unknown	—
			
	**BIOLOGICS**			
UNB	Unknown bacterial agents (non‐*Bt*)	UN/NS	Unknown	—
UNE	Botanical essence including synthetic extracts & unrefined oils	UN/NS	Unknown	—
UNF	Fungal agents	UN/NS	Unknown	—
UNM	Non‐specific mechanical disruptors	UN/NS	Unknown	—

2020 IRAC Classification.^5^, ^44^ 1988 acaricide classification 51, 52.

* NM, nerve & muscle; GD, growth & development; RSP, respiration; MG, midgut; UN/NS, unknown or non‐specific.

**Figure 5:**
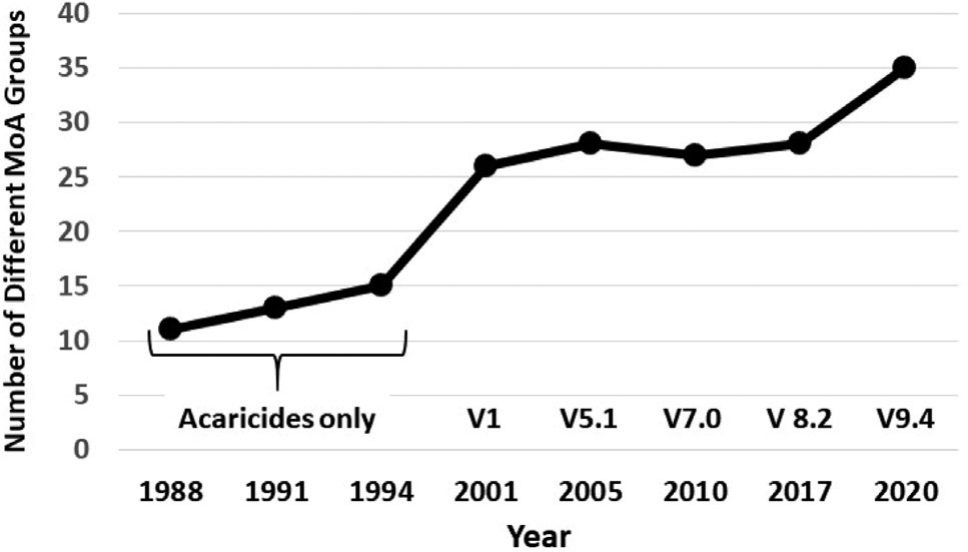
Number of different MoA classes or Groups as a function of time. 1988 to 1998 the classification was focused on acaricides. From 2001 onwards the MoA Classification Scheme included insecticides and acaricides but excluded nematicides, which are now addressed in a separate MoA Classification scheme.^5^ Between 2005^48^ and 2010^50^ continued refinement of the MoA Classification Scheme led to the removal/reclassification of some Groups resulting in a reduction in the number of Groups. Data derived, in part, from.^5^, ^48^, ^49^, ^50^, ^51^, ^52^, ^56^, ^67^

The MoA Classification Scheme has its origins in concerns regarding acaricide resistance in fruit crops during the late 1980s.^51^ *Tetranychus urticae* (two‐spotted spider mite), along with other tetranychid mites (e.g. *Panonychus ulmi*), have been one of most problematic arthropod pests in terms of resistance development for the past 40 years.^3^, ^4^, ^5^, ^9^, ^15^ The IRAC Fruit Crops WG first proposed a grouping or classification of acaricides as part of a strategy to manage spider mite resistance along with guidelines for use.^51^, ^52^, ^53^, ^54^, ^55^ In its initial form the classification was primarily focused on defining different acaricide chemical classes (Table 1), a necessarily pragmatic approach, since the knowledge of MoA and resistance and cross‐resistance mechanisms in spider mites was limited.^51^ A key premise of the recommended guidelines was the focus on rotation of acaricides.^51^, ^52^, ^55^, ^56^ Specifically, that no more than one compound from within an acaricide group should be used on the same crop during a season.^51^, ^55^ The initial acaricide classification included 11 different groups/classes (Table 1, Fig. 5) was subsequently revised and expanded in 1991^51^ (Leonard 1992) and again in 1994 as new acaricide options and information became available^55^ (Fig. 5).

As a follow‐up to the acaricide classification, in 1997 IRAC formally endorsed the concept of MoA labeling for insecticide and acaricide products as a simple, straightforward approach to provide growers, crop advisors and university personnel with information for effective rotation schemes for IRM. The acaricide classification thus evolved into a broader insecticide MoA classification in 1998, with the first official version of the Insecticide MoA Classification Scheme released in September of 2001 encompassing 26 different Groups (Fig. 5). Since then, the scheme has been continually updated with the addition of new modes of action and chemical classes^3^, ^4^, ^5^, ^48^, ^49^, ^50^, ^57^, ^58^, ^59^ (Table 1). The most up‐to‐date version can be accessed on the IRAC website.^44^ The increasing importance of the MoA Classification Scheme led to the formation of the MoA WG in 2007, which is now responsible for all updates and changes to the MoA Classification Scheme, with input from academic and industry experts in insecticide toxicology and insect biochemistry, and final review and approval by the IRAC Executive Committee.

Major changes to the scheme are indicated by a new version number (e.g. V5.0), with minor changes indicated by sub‐version (e.g. V5.3). As information on the MoA of specific insecticides and acaricides has come to light, the MoA group and subgroup listings have been revised, In some cases, in light of new information, specific insecticides have been moved into other existing groups or have been placed in entirely new MoA Groups. In a few instances some Groups (e.g. Group 27, synergists^48^ have been omitted since they no longer meet the definitions/goals of the MoA Classification Scheme. The current version (as of this writing) is V9.4 updated in March of 2020 (Table 1) encompassing 35 different groups (Fig. 5) including the multi‐site inhibitors and the newly added biologics. Some of the most recent changes such as the inclusion of biologicals and the development of a separate nematicide MoA classification scheme have recently been reviewed.^5^

#### 4.1.1 MoA labeling – a global initiative

An important result of the MoA classification scheme had been IRACs successful efforts to incorporate MoA icons (based on the MoA classification scheme) on the labels for an expanding array of products in many countries around the world. These MoA icons provide a simple mechanism for growers and crop management practitioners to identify the MoA of an insecticide product and thus facilitate rotation schemes for IRM as outlined by IRAC, country and local guidelines.

#### 4.1.2 Resistance management program for METI acaricides

During the early 1990s four new acaricides were independently developed; three by Japanese companies (fenproximate – Nihon Nohyaku, pyridaben – Nissan, tebufenpyrad – Mitsubishi) and one from the US (fenazaquin – Elanco). Fenpyroximate, pyridaben and tebufenpyrad were subsequently licensed to crop protection companies in Europe. Although all four chemistries were independently developed and had very different origin,^60^ they all shared the same MoA; inhibition of mitochondrial electron transport at complex I,^61^, ^62^ and all can adopt the same molecular shape.^60^ In an effort to mitigate potential resistance issues with these new acaricides, IRAC coordinated a resistance management program among the companies wherein only one of the four acaricides would be used each season.^55^ To further facilitate the implementation, a rapid bioassay was commissioned by IRAC,^52^, ^63^ as was a monitoring program.^55^ This program is thus an early example of IRAC facilitating a cross‐industry IRM program and benefiting from the acaricide resistance management guidelines established by IRAC^51^, ^52^ in advance of the commercialization of the METI acaricides and influenced spider mite IRM programs.^53^

### 4.2 Test methods for resistance monitoring

Resistance monitoring and documentation is central to understanding and addressing existing and developing insecticide resistance issues. Establishing baseline data for important pest insects is an essential component for resistance monitoring, ideally, with robust bioassays suitable for use across regions facilitating the reporting, sharing and straightforward comparison of resistance data. Devising field and laboratory test methods for resistance monitoring was one of the first goals of IRAC^17^ enabling resistance monitoring programs in the early 1990s.^64^ Initially overseen by a focal point within IRAC, a Test Methods WG was established (2005) to institute and harmonize protocols for bioassay methods for many of the important pest insects and mites. Today, there are more than 33 test methods that have been reviewed and validated for a wide range of pest insects and different classes of insecticides. Many of these test methods are now in the form of ‘how‐to’ videos providing a simple, easy approach on how to conduct a particular bioassay. All of these bioassay methods and videos are freely available on the IRAC website (https://irac-online.org/).

#### 4.2.1 Pollen beetle resistance monitoring

The pollen beetle (*Meligethes* spp.) is a key pest of oilseed rape in Europe with more than 515 individual cases of resistance across 27 different insecticides.^65^ A resistance monitoring program spanning 7 to 8 years, from locations across Europe was coordinated through an IRAC WG (Pollen beetle WG) tasked with the project.^66^ This program provided valuable information on the rise and spread of pyrethroid resistance in the pollen beetle across Europe and highlighted the need for IRM programs to address the spread of resistance.^66^ This research also contributed to efforts to a better understand the underlying mechanisms of the associated pyrethroid resistance,^67^, ^68^ thus further facilitating pollen beetle IRM programs. The Pollen Beetle WG subsequently evolved into the Coleoptera WG reflecting broader needs to resistance management across beetle pests.

### 4.3 Documentation of insecticide resistance – IRAC support for the APRD

Another important area of interest reflecting the original^17^ and current goals of IRAC is the facilitation of gathering and sharing of information on resistance to insecticides and insect‐resistant traits. To this end, IRAC has been a long‐term supporter^49^ of the Arthropod Pesticide Resistance Database (APRD) developed and maintained by Michigan State University (MSU).^3^, ^15^, ^65^ Since its inception in late 1990's the APRD has continued to expand with on‐going financial support from IRAC International and IRAC US. From nearly the beginning of the APRD, IRAC has had a dedicated liaison (focal point) (Fig. 2) with the MSU APRD team. Importantly, the ARPD uses the IRAC MoA Classification Scheme as the basis for its insecticide classification and as one of the search criteria in the database.^3^, ^65^

### 4.4 Position documents and guidelines

A series of position papers have been developed and are available from the IRAC website. Some of the more recent (since 2013) positions papers can be found on the IRAC website (https://irac-online.org/teams/executive/) and include:

- IRM for soil and seed applied insecticides
- Computer models applied to insecticide resistance management
- IRAC statement on combined use of chemicals and traits
- IRAC statement on IRM and the use of mixtures (see also below)
- Mixtures for insecticide resistance management in mosquito vector control
- Industry perspectives on IR monitoring
- Seed blends for IRM
- IRM in small‐holder systems

#### 4.4.1 Mixtures

Insecticide mixtures have long been viewed as one approach to resistance management^9^, ^69^, ^70^ and are an area of increasing interest as demonstrated by the precipitous rise in the number mixture‐related insecticide patents during the past decade.^71^ IRAC has addressed insecticide mixtures for crops^72^ and vector control^73^ that provides guidance regarding utility of insecticide mixtures for crop protection and resistance management.

#### 4.4.2 IRM guidelines

The different IRAC WGs have put together posters that provide guidelines for different crops and pests. Included among the crops focused documents are oilseed‐rape in Europe, corn, soybeans and cotton in Brazil, and cereals in Europe. Among the pest focused resources are IRM programs and guidelines for Colorado potato beetle, southern armyworm (S. African maize), fall armyworm (Puerto Rico), sucking insect pests, lepidoptera insect pests, thrips (*Frankliniella occidentalis*), planthoppers, cotton aphid (*Aphis gossypii*) and green peach aphid (*Myzus persicae*), mosquitoes, cockroaches, bedbugs and housefly. Also available is information on aspects of IRM biotech crops (e.g. monitoring, seed blends, combined use of insecticides and traits).

## 5 FUTURE OF IRAC

IRAC was established initially for the purposes of defining and documenting resistance, and understanding resistance mechanisms, sponsoring a number of studies (Jackson 1986, Voss 1988). The intention was for the agricultural industry to be better able to manage resistant pest populations. Over time IRAC has increasingly turned towards proactive IRM with the intention of delaying the onset of resistance, reducing its spread, and managing the economic and environmental impacts. The initiation and continued development of the MoA Classification Scheme has provided the foundation for proactive IRM based on the rotation of insecticides from different groups or subgroups. The recent extension of the MoA Classification scheme to include biologics (Table 1)^5^ provides additional support for IRM. Ensuring that the MOA classification of an insecticide is prominently displayed on the insecticide label further enables effective rotations for IRM.

Attention has turned to developing specific science‐based and practical recommendations to manage insect pests in a manner that promotes IRM, as exemplified through the use of stage^74^ or ‘windows’‐based programs^4^, ^5^, ^44^ that simplify MOA rotations that are geared to the pest and crop lifecycles. The expanding network of IRAC country teams (Figs 2, 4) provides a necessary avenue for collaborations with public sector experts, crop consultants, and extension entomologists, which are essential for developing locally appropriate IRM strategies for specific pest complexes in specific agricultural systems and public health settings.

Implementation of IRM recommendations by end‐users remains a challenge. Therefore, IRAC is also beginning to develop educational and communication tools as a means to help dealers, crop consultants and end‐users understand the potential threats of resistance and the role they can play in reducing those threats. Information is being made available via short videos, leaflets, posters, and other media, and translated into multiple languages. As part of this renewed emphasis on end‐user implementation of IRM practices, IRAC is increasing its access to the resources and connections of the CropLife International network, including their communications experts. The CropLife network reaches millions of farmers around the world with their outreach programs and has strong relationships with global institutions (such as WHO & FAO) and country regulatory agencies.

## 6 CONCLUSION

Resistance management can only be effective if there is broad cooperation among the stakeholders, including the crop protection industry, academia, regulators, crop advisors, growers and other end‐users. IRAC was established to improve resistance awareness and coordination of IRM tools and programs among crop protection product developers, forge alignment on key strategies, and provide unified communications with the other stakeholders.

As the key industry organization directed at addressing insecticide resistance, over the past 35+ years IRAC as an organization has continued to evolve and expand. The current make‐up of the participating companies in IRAC is truly global in nature and is further highlighted by the increasing number of country groups associated with IRAC (Fig. 4) which in themselves incorporate an even broader number of companies.

Thus, concerns for insecticide resistance and mitigating its impact remains of paramount importance to the companies involved in crop protection and vector control, including and especially those involved in the discovery and development of new crop protection compounds and traits. IRAC and its associated IRM guidelines and programs are all focused on maintaining the utility and efficacy of existing and new crop protection and vector control compounds, transgenic plant traits, and biological tools.

## AUTHOR CONTRIBUTIONS

TCS, RN & NS conceived the project with input from AP & RS; AP, RN, RS & NS provided background information and access to historical IRAC documents; TCS wrote the initial draft with sections added from RN & NS; All authors contributed to the editing and final version of the paper.

## CONFLICT OF INTEREST

The authors claim no conflicts of interest.
